# Understanding the Immune System’s Intricate Balance: Activation, Tolerance, and Self-Protection

**DOI:** 10.3390/ijms26125503

**Published:** 2025-06-08

**Authors:** Jui-Yun Chen, Li-Jane Shih, Min-Tser Liao, Kuo-Wang Tsai, Kuo-Cheng Lu, Wan-Chung Hu

**Affiliations:** 1 Department of Laboratory Medicine, Taipei Tzu Chi Hospital, Buddhist Tzu Chi Medical Foundation, New Taipei City 231, Taiwan; tch39410@tzuchi.com.tw; 2 Department of Medical Laboratory, Taoyuan Armed Forces General Hospital, Longtan, Taoyuan City 325, Taiwan; genelab@aftygh.gov.tw; 3 Graduate Institute of Medical Science, National Defense Medical Center, Taipei City114, Taiwan; 4 Department of Pediatrics, Taoyuan Armed Forces General Hospital, Taoyuan City 325, Taiwan; liaomintser@aftygh.gov.tw; 5 Department of Pediatrics, Tri-Service General Hospital, National Defense Medical Center, Taipei City114, Taiwan; 6 Department of Medical Research, Taipei Tzu Chi Hospital, Buddhist Tzu Chi Medical Foundation, New Taipei City 231, Taiwan; tch33225@tzuchi.com.tw; 7 Division of Nephrology, Department of Medicine, Taipei Tzu Chi Hospital, Buddhist Tzu Chi Medical Foundation, New Taipei City 231, Taiwan; tch33730@tzuchi.com.tw; 8 Department of Clinical Pathology, Taipei Tzu Chi Hospital, Buddhist Tzu Chi Medical Foundation, New Taipei City 231, Taiwan; 9 Department of Biotechnology, Ming Chuan University, Taoyuan City 333, Taiwan

**Keywords:** ACTH, steroid, IgD, gamma/delta T cells, sympathetic, parasympathetic

## Abstract

Understanding the mechanisms of immune activation and deactivation is paramount. A host must initiate effective immunity against pathogenic infections while avoiding triggering immunity against self-antigens, which can lead to detrimental autoimmune disorders. Host immunological pathways can be categorized as Immunoglobulin (Ig)G-dominant eradicable immune reactions and IgA-dominant tolerable immune reactions. Eradicable immune reactions include Th1, Th2, Th22, and Thαβ immune responses against four different types of pathogens. Tolerable immune reactions include Th1-like, Th9, Th17, and Th3 immune responses against four different types of pathogens. Here, we try to determine the mechanisms of activation and deactivation of host immune reactions. The spleen and liver play contrasting roles in mediating immune responses: the spleen is primarily involved in immune activation, whereas the liver is responsible for immune deactivation. Similarly, the sympathetic and parasympathetic nervous systems have opposing functions in immune modulation, with the sympathetic system promoting pro-inflammatory responses and the parasympathetic system facilitating anti-inflammatory processes. Furthermore, adrenocorticotropic hormone (ACTH) and glucocorticosteroids exhibit contrasting effects on immune regulation: ACTH is involved in activating adaptive immunity while inhibiting innate immunity, whereas glucocorticosteroids activate natural IgM antibody associated with innate immunity while inhibiting adaptive immunity. Heat shock proteins, particularly molecular chaperones induced by fever, play pivotal roles in immune activation. Conversely, IgD B cells and gamma/delta T cells contribute to immune deactivation through mechanisms such as clonal anergy. Understanding these mechanisms provides insights into immunological pathways, aiding in the better management of infectious diseases and autoimmune disorders.

## 1. Overview of Host Immunities

Our immune system stands as an extraordinary defense network, finely crafted to shield us from a wide spectrum of dangers while intricately managing a harmonious relationship with our own cells and tissues. At its essence, this complex system operates through two discernible mechanisms: the eradicable immune response, serving as a robust offensive against pathogens, and the tolerable immune response, representing a more controlled strategy in the face of enduring challenges [1,2,3,4]. These mechanisms coordinate a sophisticated interplay of cellular interactions, antibody generation, and regulatory measures, safeguarding our existence while mitigating the risk of autoimmune disorders [5,6].

## 2. The Eradicable Immune Response

When faced with harmful invaders, such as viruses, bacteria, parasites, or malignant cells, the body initiates its most powerful defense mechanism: the effective immune response. This comprehensive defense strategy is primarily fueled by a type of antibody called immunoglobulin G (IgG), generated by specialized B cells in collaboration with follicular helper T cells (Tfh) [1,2]. Tfh cells, identified by their expression of the CXCR5 chemokine receptor and their production of interleukin-21, are pivotal in triggering this reaction. They assist B cells within the germinal centers in transitioning from initially generating IgM antibodies to generating more potent IgG antibodies, a process facilitated by the cytokine interleukin-21. Essential transcription factors such as BCL6 and STAT5B coordinate this complex cellular process. Additionally, another subset of follicular helper T cells, Tfh13, participates in activating the Th2b immunological pathway [2,4]. Within the eradicable immune response, there are four distinct branches, each tailored to combat specific types of pathogens.

Th1 immunity combats intracellular invaders. This pathway is the body’s primary defense against intracellular microorganisms, such as certain bacteria, protozoa, and fungi. Various immune cells collaborate in this defense, including type 2 myeloid dendritic cells, type 1 innate lymphoid cells, type 1 macrophages, IFN-γ-producing CD4 T cells, CD8 T cells, type 1 invariant natural killer T cells, and IgG3 B cells [7,8]. Interleukin-12 drives this pathway, orchestrating the activation of STAT4 and STAT1 transcription factors [9]. IFN-γ—a key cytokine—activates M1 macrophages, prompting the production of free radicals. These radicals aid in killing intracellular pathogens that have been engulfed by the macrophages. This pathway is linked with type IV delayed-type hypersensitivity reactions, contributing to immune responses against intracellular invaders [10].

The Th2 immune response plays a pivotal role in defending the body against parasitic infections, which is further subdivided into Th2a and Th2b pathways. The Th2a pathway targets endoparasites, such as helminths, and involves Langerhans cells, type 2 interleukin-25-inducing innate lymphoid cells, eosinophils, mast cells, interleukin-4/5-producing CD4 T cells, iNKT2 cells, and IgG4 B cells [7,8]. The Th2b pathway focuses on ectoparasites, such as parasitic insects, and utilizes Langerhans cells, type 2 interleukin-33-inducing innate lymphoid cells, basophils, mast cells, interleukin-4/13-producing CD4 T cells, iNKT2 cells, and IgE B cells [8]. Both pathways are regulated by interleukin-4 and interleukin-5 (Th2a), interleukin-13 (Th2b), and transcription factors STAT6 and STAT1 (Th2a) or STAT3 (Th2b) [9]. They are associated with type I allergic hypersensitivity reactions, with Th2a linked to IgG4-dominant allergies and Th2b to IgE-dominant allergies [10].

The Th22 immune response actively combats extracellular pathogens. This pathway serves as the body’s frontline defense against extracellular microorganisms, including specific bacteria, fungi, and protozoa. It encompasses type 1 myeloid dendritic cells, which act as antigen-presenting cells, as well as type 3 NCR+ innate lymphoid cells, neutrophils, interleukin-22-producing CD4 T cells, and IgG2 B cells [7]. Orchestrated by interleukin-1, interleukin-6, and TNF-α, and under the regulation of STAT3 and STAT4, this pathway utilizes interleukin-22 as its primary effector cytokine [9]. Interleukin-22 activates neutrophils, enhancing processes such as phagocytosis and necroptosis, to eliminate extracellular pathogens through mechanisms such as free radical production and membrane lipid peroxidation [11]. This pathway is linked with type III immune complex-mediated hypersensitivity reactions [10,12].

Thαβ immunity defends against infectious particles. This pathway constitutes the body’s defense mechanism against infectious agents, such as viruses and prions. It involves various immune cell types, including plasmacytoid dendritic cells as antigen-presenting cells, interleukin-10-producing innate lymphoid cells, NK cells, interleukin-10-producing CD4 T cells, CD8 T cells, and IgG1 B cells. Governed by key signaling molecules, such as type 1 interferons and interleukin-10, and regulated by transcription factors STAT1, STAT2, and STAT3, this pathway employs interleukin-10 as its principal effector cytokine [9]. The primary mechanism of action involves antibody-dependent cellular cytotoxicity (ADCC), where NK cells, equipped with IgG1 antibodies, can induce apoptosis in cells infected with viruses or prions, thereby disrupting the replication and spread of these infectious agents. This pathway is linked with type II antibody-dependent cellular cytotoxic hypersensitivity reactions [3,13].

## 3. The Tolerable Immune Response

While the eradicable immune response is a potent defense mechanism, there are times when the complete eradication of a pathogen may cause severe organ damage or failure. In such situations, the body employs a more measured approach known as the tolerable immune response. This response is predominantly driven by regulatory CD4+CD25+ T cells (Treg cells) and is characterized by the production of immunoglobulin A (IgA) antibodies [1,2,4].

Treg cells, expressing the transcription factor FOXP3, secrete the cytokine TGF-β, which activates the STAT5, STAT6, and Smad transcription factors, initiating the tolerable immune response [14]. TGF-β also plays a crucial role in inducing B cells to undergo class-switching from IgM to IgA antibodies [15]. Other key players in this response include regulatory dendritic cells, regulatory B cells, and regulatory innate lymphoid cells.

Similar to the eradicable immune response, the tolerable immune response can be further subdivided into four distinct pathways:

Th1-like immunity tolerates intracellular pathogens. This pathway is the body’s tolerable response to intracellular microorganisms, such as certain bacteria, fungi, and protozoa. It involves type 2 myeloid dendritic cells as antigen-presenting cells, type 1 non-cytotoxic innate lymphoid cells, macrophages, IFN-γ/TGF-β-producing CD4 T cells, CD8 T cells, iNKT1 cells, and IgA1 B cells [7]. Driven by interleukin-12 and TGF-β, this pathway is associated with type IV delayed-type hypersensitivity reactions [9].

Th9 immunity tolerates parasites. This pathway is the body’s tolerable response to parasitic infections, encompassing both endoparasites (helminths) and ectoparasites (insects). It involves Langerhans cells as antigen-presenting cells, TSLP-inducing type 2 innate lymphoid cells, regulatory eosinophils, basophils, mast cells, interleukin-9-producing CD4 T cells, iNKT2 cells, and IgA2 B cells [7,16]. Driven by interleukin-4 and TGF-β, this pathway is associated with type I allergic hypersensitivity reactions [9,17].

Th17 immunity tolerates extracellular pathogens. This pathway is the body’s tolerable response to extracellular microorganisms, such as certain bacteria, fungi, and protozoa [18,19]. It involves type 1 myeloid dendritic cells as antigen-presenting cells, type 3 non-cytotoxic innate lymphoid cells, neutrophils, interleukin-17-producing CD4 T cells, iNKT17 cells, and IgA2 B cells [7,8]. Driven by interleukin-6 and TGF-β, this pathway is associated with type III immune complex-mediated hypersensitivity reactions [9,10,20].

Th3 immunity tolerates infectious particles. This pathway is the body’s tolerable response to infectious particles, such as viruses and prions. It involves plasmacytoid dendritic cells as antigen-presenting cells, interleukin-10-producing innate lymphoid cells, NK cells, interleukin-10/TGF-β-producing CD4 T cells, CD8 T cells, and IgA1 B cells. Driven by TGF-β and interleukin-10, and governed by STAT1, STAT2, STAT3, and STAT5, this pathway is associated with type II antibody-dependent cellular cytotoxic hypersensitivity reactions.

## 4. Immune Activation and Deactivation Mechanisms

### 4.1. The Spleen and Liver: Opposing Roles in Immune Regulation

The spleen and liver are two crucial abdominal organs that play opposing roles in immune activation and deactivation. Splenectomy (i.e., the removal of the spleen) is sometimes performed in severe autoimmune hematological disorders, such as immune thrombocytopenia. However, this procedure can leave patients more susceptible to infections, especially those caused by encapsulated bacteria, such as Streptococcus, meningococcus, and Hemophilus.

The spleen is a key organ for immune activation, producing IgM antibodies against bacterial infections. Within the spleen, the white pulp contains B lymphocyte germinal centers, while the surrounding red pulp houses macrophages that digest old red blood cells and platelets. When bacterial antigens enter the lymph or blood drainage to the spleen, the organ can sense these antigens and activate B cells to produce IgM antibodies.

Immature B lymphocytes are formed in the bone marrow and enter the spleen for further maturation, potentially becoming spleen marginal zone B-2 lymphocytes that produce T cell-independent IgM antibodies against bacterial polysaccharides or glycolipid antigens. This explains why splenectomy can increase the risk of infections by encapsulated bacteria with polysaccharide antigens. In the spleen’s white pulp, follicular B-2 lymphocytes also react to T cell-dependent antigens to produce IgM and later IgG antibodies. In the red pulp, macrophages provide additional immune stimulation. The degraded products of digested red blood cells, including hemoglobin and heme constituents, act as potent immune stimulants, aiding in the maturation and activation of B-2 cells to produce IgM antibodies [21,22,23]. The spleen is also home to resident memory IgM B cells and abundant heme oxygenase (HO-1) in its macrophages [23]. Heme oxygenase (HO-1) catalyzes the breakdown of heme into ferrous iron, carbon monoxide, and biliverdin, which readily becomes bilirubin [24,25]. These end products (carbon monoxide, biliverdin, and bilirubin) act as immunosuppressants, with carbon monoxide exhibiting anti-inflammatory properties in contrast to the immune-stimulating effects of nitrogen monoxide generated by induced nitric oxide synthase (iNOS) [26].

After leaving the spleen macrophages, bilirubin has a strong affinity for serum albumin and is transported to the liver through the spleen vein. Along with IgM antibodies generated from the spleen’s white pulp, these molecules enter the liver’s portal vein system, joining the inferior mesenteric vein and superior mesenteric vein, which carry digested food constituents from the stomach and intestines.

Once in the liver, the portal veins become sinusoid vessels containing Kupffer macrophages. These cells digest antigen–IgM antibody complexes, neutralizing bacterial pathogens that may have contaminated food contents. They also clear antigen–IgG antibody complexes produced by spleen follicular B-2 cells, explaining the hypogammaglobulinemia observed in chronic liver disease with Kupffer cell dysfunction. B-1 cells, which produce natural T cell-independent IgM antibodies, can also be found in fetal liver, peritoneum, and mucosal sites.

Safe, digested food contents are not engulfed by Kupffer cells but metabolized into useful molecules within the liver. The liver also contains γδ T cells that mediate clonal anergy, a process that will be discussed later. Additionally, unconjugated bilirubin becomes water-soluble and is conjugated in the liver, aiding in the process of clonal anergy to food molecules.

Thus, the liver serves as the main organ inducing food antigen tolerance, preventing immune reactions to common food components, such as small peptides or glycolipids. Bilirubin, with its immunosuppressive effects, can be stored in the gallbladder and later secreted into the intestine, further contributing to the clonal anergy of intestinal γδ T cells against food antigens.

Most conjugated bilirubin in the large intestine is metabolized by intestinal bacteria into urobilinogen, which can be reabsorbed and re-secreted through enterohepatic circulation. Bilirubin’s immunosuppressive effects may explain why liver failure patients with elevated bilirubin levels have higher incidences of sepsis, as its normal physiological function involves immune modulation.

**Figure 1:**
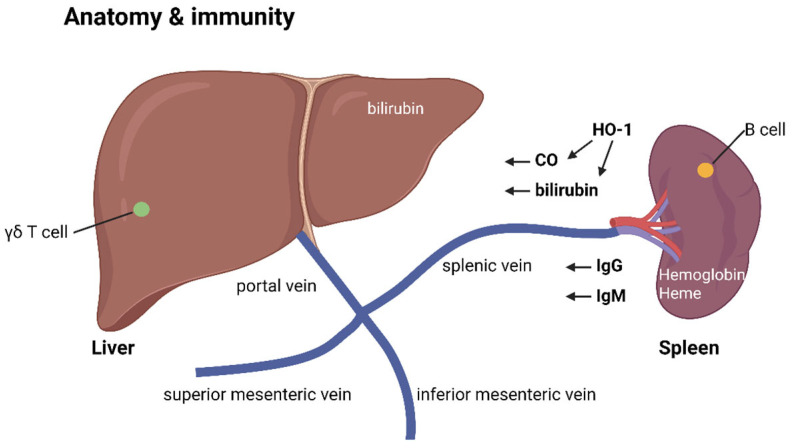
The immune activation and tolerance mechanisms in the liver and spleen. The spleen is responsible for generating B cell antibodies, including IgG and IgM, and the liver is responsible for generating gamma/delta T cells with clonal anergy. Heme production from hemoglobin inside the spleen provides an immune activation environment in the spleen. The spleen HO-1 enzyme generates CO and bilirubin transported via the splenic vein to the liver to help immune tolerance in the liver.

The intestine also plays a role in mucosal immune reactions. The Peyer’s patch in the intestine allows B lymphocytes to produce tolerable IgA antibodies against bacterial antigens from the intestinal tract. The resulting IgA–bacterial antigen complexes do not enter the inferior mesenteric vein or superior mesenteric vein, preventing an increase in IgA levels in the portal vein. The relationship between immune regulation and the spleen/liver is shown in Figure 1.

### 4.2. The Autonomic Nervous System: The Balance of Inflammation and Anti-Inflammation

The autonomic nervous system, with its two major components, the sympathetic and parasympathetic branches, plays a vital role in immune regulation, akin to a skilled musician fine-tuning the resonance of a stringed instrument [27,28]. The sympathetic nervous system, fueled by the neurotransmitters dopamine, epinephrine, and norepinephrine, strikes a pro-inflammatory chord, amplifying the body’s defensive response [29,30,31,32,33,34].

Dopamine, in particular, takes center stage as the key mediator in triggering follicular helper T cells, the initiators of eradicable host immune responses [35]. Like a virtuoso conductor, dopamine orchestrates the cellular interactions that culminate in the production of IgG antibodies against invading pathogens. Norepinephrine is the next synthesized molecule after dopamine, and it has the potential to promote Thαβ anti-viral immunity while suppressing Th1 anti-intracellular microorganism immunity [36,37,38]. Finally, epinephrine has the potential to promote Th22/Th17 anti-bacterial immunity while suppressing Th2 anti-parasite immunity [39,40,41,42,43,44,45]. The sympathetic nerve system is activated in emergent situations, such as viral or extracellular bacterial infections. Thus, Thαβ and Th22/Th17 immunities are upregulated in sympathetic nerve system activation while sacrificing more chronic Th1 or Th2 immunities.

The sympathetic nervous system further influences the immune response, promoting the Th1, Th2, Th17, and Thαβ pathways [34,46,47,48]. However, like all great compositions, there exists a delicate counterpoint—a negative feedback loop that tempers the exuberance of autocrine catecholamines, preventing unchecked lymphocyte proliferation [27,49]. In contrast, the parasympathetic nervous system, with its cholinergic nerves, takes on the role of tempering the inflammatory fervor with its anti-inflammatory effects. The cholinergic anti-inflammatory pathway acts as the efferent, or motor arm, of the inflammatory reflex, the neural circuit that responds to and mediates the inflammatory response [50].

The vagus nerve, part of the tenth cranial nerve, supplies nerves to the celiac ganglion, where the splenic nerve originates. Its outgoing stimulation reduces the heart rate, promotes gastrointestinal movement, and notably, suppresses the production of the pro-inflammatory cytokine TNFα in the spleen.

The neurotransmitter acetylcholine, released by the efferent pathway of the vagus nerve, acts as a soothing soloist, interacting with the α7 subunit of the nicotinic acetylcholine receptor (α7 nAChR) expressed on the cell membranes of macrophages and other cytokine-secreting cells. Through this intricate mediation of molecular interactions, acetylcholine activates intracellular signal transduction pathways, effectively reducing the release of pro-inflammatory cytokines [51].

Acetylcholine further influences the immune response, inhibiting the Th1, Th2, Th17, and Thαβ pathways in a harmonious counterpoint to the pro-inflammatory melodies of the sympathetic nervous system [52,53]. Moreover, acetylcholine engages in an augmented interaction with TGFβ, a potent immunomodulatory cytokine for initiating tolerable immunities, further reinforcing its role in reducing immunity [54,55].

It is worth noting that the opposing forces of the sympathetic and parasympathetic nervous systems do not create discord but rather represent a delicate interplay, a harmonic balance of the immune response. The sympathetic nerve employs cyclic AMP (cAMP) as its intracellular second messenger, while the parasympathetic nerve utilizes cyclic GMP (cGMP) [56,57,58]. Though both are derived from purines, the direction of their NH2 groups is opposite, with opposite molecular function.

This natural competition between cAMP and cGMP, mediated by their respective effector proteins, protein kinase A and protein kinase G, governs the intricate interplay between the two autonomic branches [59,60,61]. The substrate amino acid sequence consensus motifs for these kinases, while sharing similarities, bear subtle distinctions, causing different cellular functions.

**Figure 2:**
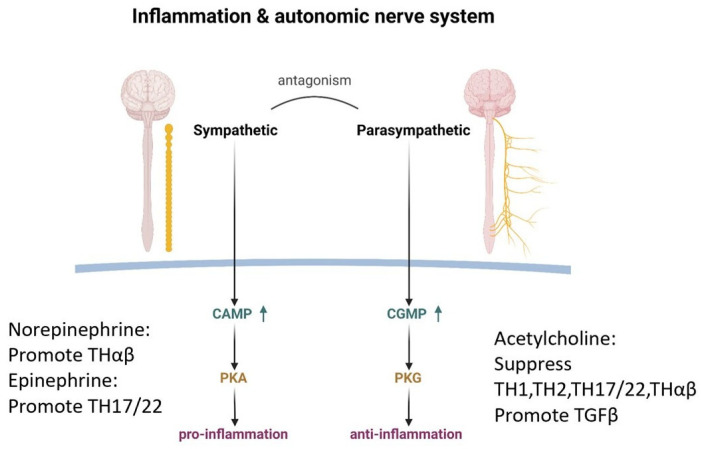
The relationship between the autonomic nerve system and inflammation and anti-inflammation. The sympathetic nerve will enhance intracellular cAMP to activate protein kinase A to induce inflammation. Norepinephrine can promote THαβ immunity, and epinephrine can promote TH17/22 immunity. The parasympathetic nerve will enhance intracellular cGMP to activate protein kinase G to suppress inflammation. Acetylcholine can suppress TH1, TH2, TH17/22, and THαβ immunities and promote TGFβ activity.

Ultimately, the sympathetic nerve system, with its pro-inflammatory “fear or fight” response, and the parasympathetic nerve system, with its anti-inflammatory “rest and digest” response, strike a delicate harmony, regulating the body’s inflammatory response with the precision of a finely tuned orchestra. The relationship between immune regulation and the autonomic nerve system is shown in Figure 2.

### 4.3. The Endocrine System: Modulating the Immunity

The endocrine system, through the intricate interplay of hormones, modulates the tempo and intensity of the immune response. At the center lies the hypothalamic–pituitary–adrenal (HPA) axis, which orchestrates the delicate balance between inflammation and immunosuppression.

The hypothalamus initiates the mediation by secreting corticotropin-releasing hormone (CRH), a signal that stimulates the pituitary gland to release adrenocorticotropic hormone (ACTH). ACTH, in turn, prompts the adrenal cortex to secrete glucocorticosteroids, the potent immunosuppressants that form the backbone of the stress response.

Glucocorticosteroids exert a profound influence on the immune system. Their primary function is immunosuppression, a carefully modulated restraint that prevents the body’s defensive forces from unchecked autoimmunity. However, glucocorticosteroids exhibit the dual effect of inhibiting adaptive immunity while concurrently enhancing the body’s innate defense mechanisms to upregulate natural T cell-independent IgM antibodies from B-1 cells or spleen marginal zone B-2 cells [62,63,64]. This enables the body to swiftly initiate a nonspecific response to acute infections, particularly encapsulated bacterial threats, by increasing the presence of neutrophils and other innate immune cells [65]. This can explain why glucocorticosteroids are effective in treating encapsulated bacterial infections, such as Streptococcus pneumonia and Hemophilus influenza infections.

However, this shift in emphasis comes at a cost, namely the profound suppression of lymphocytes, the very cells that orchestrate the body’s adaptive, antigen-specific defenses. This is a trade-off or a strategic sacrifice of specificity for the sake of immediate survival.

Yet, there exists a delicate balance, a counterpoint that prevents the overactivity. The HPA axis employs a negative feedback loop. Increased glucocorticosteroid levels suppress the production of ACTH, modulating the intensity of the stress response.

The interplay between ACTH and glucocorticosteroids is itself a delicate counterpoint that balances the immune response. While glucocorticosteroids suppress adaptive immunity, ACTH stimulates lymphocyte activity [66,67]. This opposing force is an intricate balance of the endocrine system’s influence on immunity.

ACTH’s influence extends further, inhibiting Th17-like innate immune reactions, a subtle modulation akin to a skilled composer introducing unexpected harmonic progressions [68,69,70,71]. This delicate interplay between ACTH and glucocorticosteroids, representing a Yin–Yang regulation of endocrine-mediated immune control, ensures that the body’s defensive forces remain poised, neither over-reacting nor hypo-reacting.

While CRH, ACTH, and glucocorticosteroids form the core ensemble of the HPA axis, the performance is enriched by the contributions of other cytokines. CRH can induce innate immunity through the upregulation of pro-inflammatory cytokines, a stirring prelude that sets the stage for the ensuing immune response [72,73,74,75].

**Figure 3:**
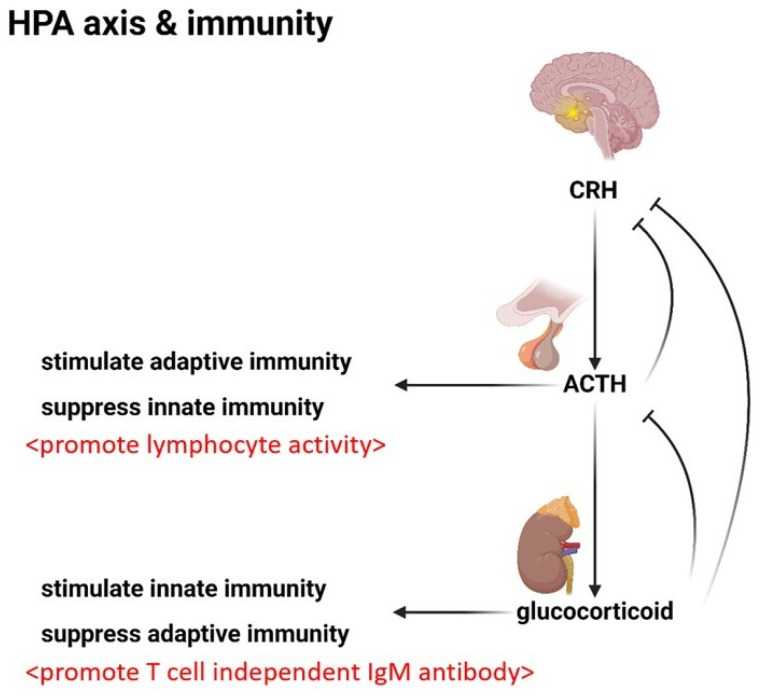
The HPA axis and immunity. The HPA axis contains the CRH -> ACTH -> glucocorticoid pathway. There is also negative feedback. ACTH can inhibit CRH, and glucocorticoids can inhibit CRH as well as ACTH. ACTH can suppress innate immunity and stimulate adaptive immunity by promoting lymphocyte activity. Glucocorticoids can suppress adaptive immunity and stimulate innate immunity by promoting T cell-independent IgM antibody.

However, CRH’s influence is primarily restricted to the central nervous system; its role in the systemic circulation is less pronounced than that of its hormonal counterparts, ACTH and glucocorticosteroids. Figure 3 shows the HPA axis and its relationship to immunity.

### 4.4. Immune Activation: The Mechanism

**Figure 4:**
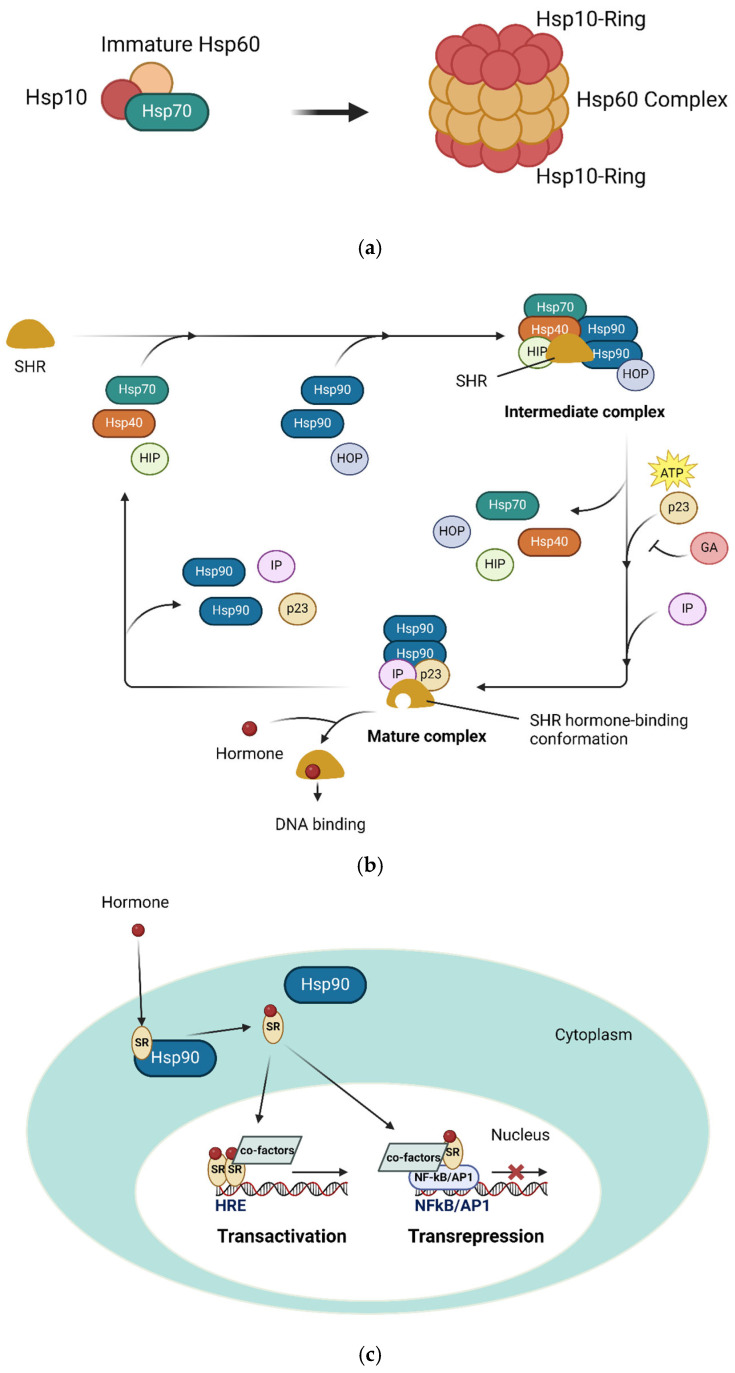
(**a**) HSP70 and HSP60/HSP10 complex; (**b**) HSP70/40 and HSP90/SR; (**c**) steroid receptor. HSP70 can help generate a mature HSP60/HSP10 complex. HSP40, HSP70, HSP90, and SHR can also form an intermediate complex. Then, a mature complex with HSP90 and SHR can be formed to inhibit the activity of SHR. When steroid binds to SHR in the HSP90/SHR complex, the SHR will be released from the HSP90/SHR complex to bind to DNA for HRE transactivation and NFkB/AP1 transrepression.

When the body encounters a pathogenic threat, a signal is sent that mobilizes the intricate machinery of the immune system. This call to arms takes the form of a fever, a carefully orchestrated rise in core body temperature above the normal 37 °C. This is a strategic maneuver that activates a crucial ensemble of molecular players: heat shock proteins. At the cellular level, heat shock proteins trigger a cascade of defensive responses that culminate in the generation of adaptive immunity, including the production of vital antibodies [76]. These molecular chaperones, including HSP60 and HSP70, bind to newly synthesized proteins, facilitating their proper folding and ensuring the generation of suitable antigens to trigger the adaptive immune response. HSP70 can help HSP60 and HSP10 form a barrel-like structure, which aids in protein folding (Figure 4a). HSP70 also helps to translocate unfolded proteins. Chaperon-assisted protein folding requires ATP energy and occurs in special situations. The majority of proteins are folded spontaneously in normal situations.

In the face of viral invasion, the heat shock proteins of the host cell bind to the newly synthesized viral proteins, ensuring their proper three-dimensional structure independent of the viral nucleic acids. This strategic maneuver sets the stage for the next critical step—the activation of the immunoproteasome, a specialized molecular complex that digests pathogenic proteins, generating the precise peptide antigens necessary for antigen presentation, T cell recognition, and ultimately, antibody production.

The heat shock proteins’ influence extends beyond mere chaperoning, assuming a strategic role in regulating the body’s hormonal defenses. HSP90, once activated, binds to steroid receptors, preventing their interaction with glucocorticosteroids and their subsequent binding to target DNA. Before the formation of the mature HSP90 and steroid receptor complex, HSP70 and HSP40 assist in forming an immature complex that promotes the final formation of the mature complex [77] (Figure 4b,c). This effectively short-circuits the suppressive effects of steroid signaling on adaptive immunity, allowing the body’s defensive forces to mount an unencumbered response.

Moreover, heat shock proteins can bind to Toll-like receptors, initiating a cascade of signaling events that stimulate the host’s immune response against diverse pathogens. This multifaceted role, encompassing chaperoning, antigen processing, and immunomodulation, solidifies the heat shock proteins’ position as the indispensable roles of immune activation.

### 4.5. Antibody-Dependent Enhancement: An Adverse Effect

While the immune system’s intricate mechanism is a marvelous design, there is a phenomenon known as antibody-dependent enhancement (ADE). In several viral infections, the very antibodies meant to neutralize the pathogen can paradoxically exacerbate the disease, a perplexing and potentially dangerous scenario.

The underlying mechanisms of ADE have been the subject of intense debate, with hypotheses including antibody uptake by Fc receptors and complement receptor-mediated uptake by monocytes [78,79,80]. However, a compelling theory has emerged that highlights the critical importance of eliciting the appropriate antibody response in the development of effective vaccines against viral pathogens [81].

This theory lies in the observation that not all antibodies are created equal in their ability to counter viral threats. While IgG1 antibodies are the body’s primary defense against viral invaders, other antibody isotypes, such as IgG3, can inadvertently aid the pathogen’s entry and propagation within host cells [82].

When monocytes or macrophages encounter these non-neutralizing antibodies bound to viral particles, they engulf the complexes but cannot destroy them. Once within these immune cells, the virus can replicate unimpeded, triggering a cascade of pro-inflammatory cytokines that can exacerbate the disease’s severity.

This cautionary example underscores the critical importance of eliciting the appropriate IgG1 antibody response in the development of effective viral vaccines. The Thαβ immune pathway, distinct from the traditional Th1 pathway, is the body’s primary defense against viral pathogens, and it drives the production of the coveted IgG1 antibodies.

In contrast, the Th1 pathway, while against intracellular bacterial and protozoal threats, predominantly elicits IgG3 antibodies, which may inadvertently contribute to ADE in the context of viral infections. This is a subtle yet critical distinction that highlights the intricate immune system.

**Figure 5:**
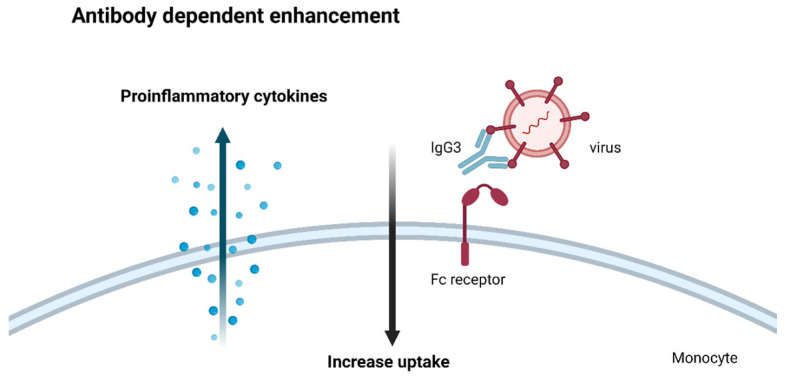
Antibody-dependent enhancement. The IgG3 antibody is mainly against intracellular bacteria. When a virus infection induces IgG3 antibody generation, it can bind to the virus and the Fc receptor to be ingested by macrophages. Macrophages cannot digest virus particles but instead produce a lot of pro-inflammatory cytokines to induce a cytokine storm. This is the mechanism of antibody-dependent enhancement.

To develop truly effective vaccines against viral threats, researchers must carefully elicit the appropriate antibody response through the precise modulation of the Thαβ pathway to avoid ADE. There is an alternate hypothesis saying that ADE is due to different serotypes of certain viruses [83]. However, this cannot explain why pro-inflammatory cytokines are uncontrolledly upregulated during ADE. This hypothesis cannot successfully explain the roles of monocytes or macrophages in ADE. The ADE mechanism is shown in Figure 5.

### 4.6. Clonal Anergy: The Art of Self-Tolerance

**Figure 6:**
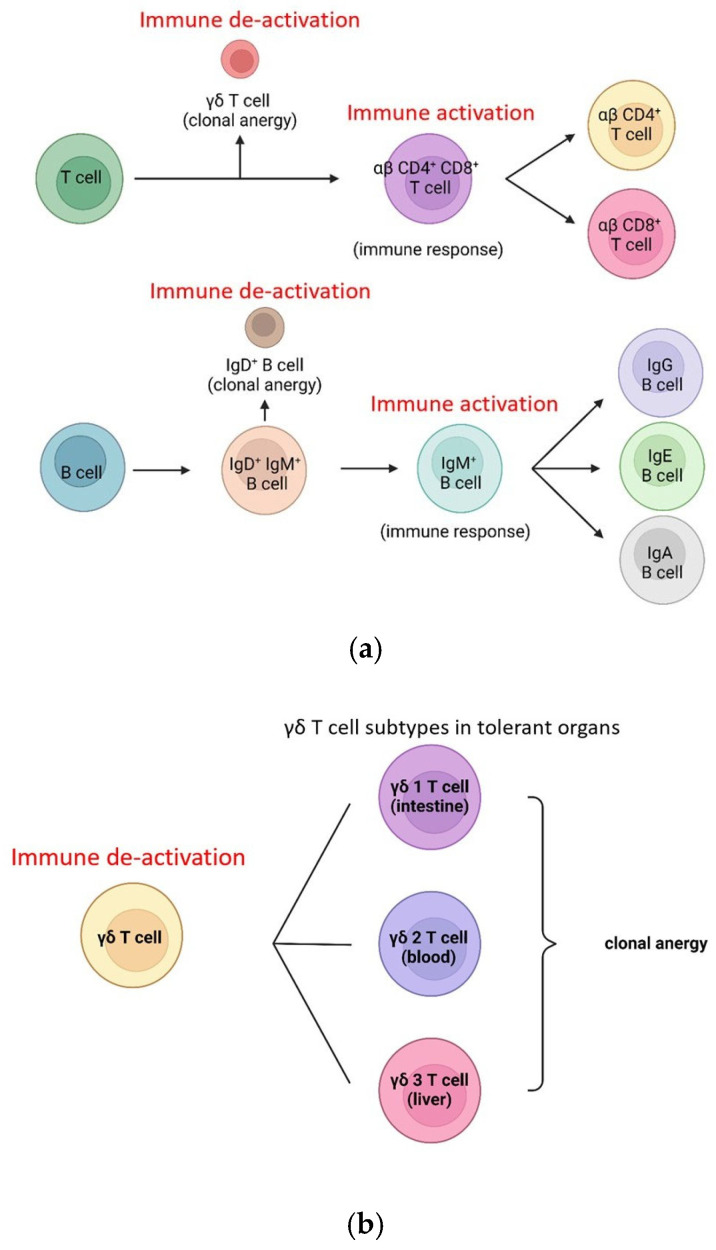
(**a**) Gamma/delta T cells and IgD B cells; (**b**) classification of gamma/delta T cells. The immune deactivation is related to the clonal anergy of gamma/delta T cells and to the clonal anergy of IgD B cells. The immune activation is related to the IgM B cells and subsequent IgG/IgE/IgA B cells and to the alpha/beta T cells. Gamma/delta T cells have three subtypes that exist in the intestine, blood, and liver to mediate immune tolerance.

The body’s ability to distinguish self from non-self is a delicate mechanism that prevents the immune system from mounting an unnecessary and potentially devastating response against its own tissues. This intricate machinery, known as clonal anergy, is orchestrated by two distinct cell types: gamma/delta (γδ) T cells and IgD B cells, acting as the sentinels of self-tolerance (Figure 6a).

The gamma/delta T cells take center stage early in the immune response, developing in the thymus before the emergence of their more abundant alpha/beta (αβ) counterparts [84]. During this formative stage, if a T cell clone recognizes a self-antigen, it is not silenced but rather redirected, differentiating into a gamma/delta T cell. This strategic maneuver effectively prevents the subsequent development of alpha/beta T cells that could potentially mount an autoimmune response against the body’s own tissues [85,86,87,88,89,90,91,92]. Circulating gamma/delta T cells can prevent the false activation of alpha/beta T cells toward self-antigens. In addition to blood, gamma/delta T cells are located in the intestine and liver, which are immune-tolerant organs (Figure 6b). The TCR signaling pathways of alpha/beta T cells and gamma/delta T cells differ, and conformational changes in gamma/delta T cell CD3 cannot be triggered by antigens [93,94,95,96,97,98]. There is an alternate hypothesis saying gamma/delta T cells are responsible for innate immunity [99,100]. However, this hypothesis cannot explain why gamma/delta T cells must be generated in the thymus earlier and exclusively than alpha/beta T cells.

B lymphocytes employ a similar mechanism to maintain self-tolerance [101]. Mature B cells co-express IgD and IgM antibodies on their surface, each isotype serving a distinct purpose in the intricate machinery of immunity [102]. If a self-antigen is recognized by the IgD antibody, it triggers clonal anergy, effectively preventing an immune response against the body’s own molecules [103,104,105]. The immune function of IgD was proposed, but IgD neutralization did not show any immune defects against pathogens but suppressed autoimmunity [106]. Thus, clonal anergy is the better machinery of IgD B cells.

However, should a foreign antigen be detected by the IgM antibody, a different sequence unfolds that initiates a robust immune response against the pathogenic invader [107]. This strategic distinction allows B cells to undergo antibody isotype class switching, producing IgG, IgE, or IgA antibodies tailored to the specific threat, and further refining the body’s defensive arsenal. Gamma/delta T cells can also aid in B cell tolerance [108].

This intricate system of checks and balances, orchestrated by gamma/delta T cells and IgD B cells, represents a masterful composition, a delicate interplay of cellular choreography and molecular recognition that ensures the immune system remains vigilant against external threats while maintaining a delicate tolerance toward the body’s own tissues.

The remarkable intricacy of the human immune system fulfills a vital role in safeguarding us from harm while preserving the essential balance necessary for our survival. The whole immunological framework is summarized in Table 1.

## 5. Conclusions

The intricate coordination of the human immune system is in perfect harmony. Notably, the spleen and liver play contrasting roles in initiating immune responses and promoting immune tolerance. The autonomic nervous system, comprising its sympathetic and parasympathetic branches, plays a pivotal role in orchestrating immune activation and deactivation. These opposing forces collaborate in a finely tuned balance, ensuring the delicate equilibrium between protective responses and tolerance. Similarly, the endocrine system, mediated by the intricate interplay of the HPA axis and its hormonal components, regulates the magnitude and pace of the immune response. This coordination guarantees that the body’s defenses remain vigilant, avoiding exaggerated reactions to harmless stimuli while safeguarding against the dangers of autoimmune disorders. At the cellular level, numerous molecular participants assume crucial roles, ranging from heat shock proteins, which mobilize the immune response against pathogens, to gamma/delta T cells and IgD B cells, which intricately regulate self-tolerance. Each element, cellular interaction, and signaling pathway contributes significantly to the overall orchestration of immunity.

From a future perspective, we can use the immune activation and deactivation to deal with numerous types of pathogen infections as well as autoimmune diseases. We can trigger protective immune reactions against the four types of pathogens via the mechanism of immune activation. We can also use immune deactivation machinery to avoid detrimental autoimmune disorders. These strategies will be very helpful in managing or treating clinical illnesses.

## Figures and Tables

**Table 1:** Summary of host immunological pathways.

Immune Pathways	Driven Cytokines, ILCs, DC	Transcription Factors	Effector Cells	CD4 T Cells	B Cells	NKT Cells	Pathogen/Pathogenesis	Autoimmune
Initiatory								
Tfh	IL-21, FDC, LTi	STAT1, STAT3, STAT5B		IL-21 CD4 T cells	IgG B cells	iNKTfh		
Eradicable immunities								
TH1	IL-12, ILC1, mDC2	STAT4	Macrophages (M1), CTL (Tc1, EM4)	IFN-γCD4 T cells	IgG3 B cells	iNKT1	Intracellular microorganisms (bacteria, fungi, and protozoa)	Type 4 DTH
TH2 (TH2a)	IL-4, iILC2, LC	STAT6, STAT1	Eosinophils (iEOS), mast cells (MCt)	IL-4, IL-5 CD4 T cells	IgG4 B cells	iNKT2	Endoparasites (helminths)	Type 1 allergy (IgG4)
TH2 (TH2b)	IL-4, nILC2, LC, Tfh13	STAT6, STAT3	Basophils, mast cells (MCct)	IL-4, IL-13 CD4 T cells	IgE B cells	iNKT2	Ectoparasites (Insects)	Type 1 allergy (IgE)
TH22	IL-1, mDC1, ILC3 NCR+	STAT3	Neutrophils (N1)	IL-1, TNFα, IL-22 CD4 T cells	IgG2 B cells	iNKT17	Extracellular microorganisms (bacteria, fungi, and protozoa)	Type 3 Immune complex
THαβ	IL-10, pDC, IFNα, ILC10	STAT1, STAT2	NK cells (NK1), CTL (Tc2, EM1)	IL-10 CD4 T cells	IgG1 B cells	iNKT10	Infectious particles (viruses/prions)	Type 2 ADCC
**Immune pathways**	**Driven cytokines, ILCs**	**Transcription factors**	**Effector cells**	**CD4 T cells**	**B cells**	**NKT cells**	**Pathogen/pathogenesis**	**Autoimmune**
Regulatory								
Treg	TGFβ, DCreg, ILCreg	STAT5A, STAT5B		TGF-β CD4 T cells	IgA B cells	iNKTreg		
Tolerable immunities								
TH1like	IL-12, TGF-β, ILC1	STAT4, STAT5	Macrophages (M2), CD8 T cells (EM3)	IFN-γ/TGF-β CD4 T cells	IgA1 B cells	iNKT1	Intracellular microorganisms (bacteria, fungi, and protozoa)	Type 4 DTH
TH9	IL-4, TGF-β, TSLP ILC2	STAT6, STAT5	Eosinophils (rEOS), basophils, mast cells (MMC9)	IL-9 CD4 T cells	IgA2 B cells	iNKT2	Parasites (helminths and insects)	Type 1 allergy
TH17	IL-6, TGF-β, ILC3 NCR-	STAT3, STAT5	Neutrophils (N2)	IL-17 CD4 T cells	IgA2 B cells	iNKT17	Extracellular microorganisms (bacteria, fungi, and protozoa)	Type 3 immune complex
TH3	IL-10, TGF-β, ILC10	STAT1, STAT2, STAT5	NK cells (NK2), CD8 T cells (EM2)	IL-10/TGF-β CD4 T cells	IgA1 B cells	iNKT10	Infectious particles (viruses/prions)	Type 2 ADCC

## Data Availability

Data sharing is not applicable to this article as no new data were created or analyzed in this study.
